# *Streptococcus pneumoniae* serotypes and factors associated with antimicrobial resistance in Invasive pneumococcal disease cases in Latvia, 2012–2022

**DOI:** 10.3389/fpubh.2025.1501821

**Published:** 2025-03-12

**Authors:** Larisa Savrasova, Anita Villerusa, Indra Zeltina, Angelika Krumina, Hedija Cupeca, Sooria Balasegaram, Mara Greve, Oksana Savicka, Solvita Selderina, Jelena Galajeva, Diana Dushacka

**Affiliations:** ^1^Institute of Public Health, Riga Stradinš University, Riga, Latvia; ^2^Riga East University Hospital Riga, Riga, Latvia; ^3^Department of Infectology, Riga Stradiņš University, Riga, Latvia; ^4^Children’s Clinical University Hospital, Riga, Latvia; ^5^Department of Human Physiology and Biochemistry, Riga Stradiņš University, Riga, Latvia; ^6^Public Health England Field Epidemiology Service South East and London, London, United Kingdom; ^7^Statistics Unit, Riga Stradiņš University, Riga, Latvia; ^8^Laboratory “Latvian Centre of Infectious Diseases”, National Microbiology Reference Laboratory, Riga, Latvia

**Keywords:** *Streptococcus pneumoniae*, Invasive pneumococcal disease (IPD), serotype replacement, antimicrobial resistance (AMR), pneumococcal conjugate vaccines (PCV), surveillance, Latvia

## Abstract

**Background:**

*Streptococcus pneumoniae* is a major cause of Invasive pneumococcal disease (IPD), including bacteremic pneumonia, septicemia, and meningitis. The introduction of pneumococcal conjugate vaccines (PCVs) has significantly reduced the incidence of IPD caused by vaccine-covered serotypes. However, serotype replacement and antimicrobial resistance remain concerns. In Latvia, vaccination against pneumococcal disease was introduced into the NIP in 2010 with PCV7, later transitioning to PCV10 in 2012 and to PCV15 in 2024. This study aims is to determine the changes in *S. pneumoniae* antimicrobial resistance and its association with PCV10 serotypes in Latvia.

**Materials and methods:**

We conducted a population-based cross-sectional study using IPD surveillance data from Latvia over an 11-year period (2012–2022). IPD cases were defined according to the European Union case definition. Serotyping and antimicrobial susceptibility testing were performed on isolates from normally sterile sites. We analyzed the differences in IPD incidence, serotype distribution, and antimicrobial resistance using chi-square tests and multivariable logistic regression was used to determine associations between antimicrobial resistance and risk factors.

**Results:**

A total of 811 IPD cases were reported, with significant differences observed across the study period (*p* < 0.001). The most common serotypes were 3 and 19A. The proportion of IPD cases caused by PCV10 serotypes significantly decreased over the years, while cases caused by PCV13, PCV15, and PPPV23 serotypes increased. Antimicrobial susceptibility testing revealed resistance rates of 3.8% to penicillin, 5.4% to erythromycin, and 1.2% to cefotaxime/ceftriaxone. Erythromycin resistance showed significant variation over time (*p* = 0.016), decreasing from 7.1% in 2012 to 4.8% in 2022. Multivariable logistic regression indicated that IPD cases with *S. pneumoniae* PCV10 serotypes and meningitis were significantly associated with an increased likelihood of penicillin and erythromycin resistance.

**Conclusion:**

The study highlights a decrease in erythromycin resistance in IPD cases over time and significant associations between PCV10 serotypes and meningitis in IPD cases and penicillin and erythromycin resistance. The findings underscore the importance of continuous surveillance of *S. pneumoniae* serotypes and antimicrobial resistance patterns to inform treatment guidelines and vaccination policies. Further research is needed to assess the long-term impact of the PCV15 vaccine on *S. pneumoniae* serotype distribution and resistance.

## Introduction

*Streptococcus pneumoniae* is a bacterium responsible for Invasive pneumococcal disease (IPD), with bacteremic pneumonia, septicemia, and meningitis as the most common clinical presentations (1, 2). In Europe, the mean annual incidence of IPD in children under 2 years of age before the introduction of PCV7 was 44.4 per 100,000 population (3). In Latvia, the highest annual IPD incidence recorded during the PCV10 vaccination era (2012–2018) was 4.4 per 100,000 population in 2015, with infants and the older adult being the most affected groups (4).

The highest IPD burden is in infants, older adults, and those with certain comorbidities (5). Considering the IPD burden is important because pneumococcal conjugate vaccines are currently in almost all European countries’ national immunization programs (1).

The introduction of pneumococcal conjugate vaccines (PCV) into the National Immunization Programs (NIPs) across Europe, including Latvia, has significantly reduced the incidence of IPD caused by vaccine-included serotypes. However, the phenomenon of serotype replacement has been observed, with non-vaccine serotypes, such as 15B, 12F, 3, 17F, and 19A, becoming more prevalent (6–9). This serotype replacement presents a challenge to the continued effectiveness of current vaccination strategies.

Additionally, the introduction of PCVs has influenced *S. pneumoniae* antimicrobial resistance patterns (10). For instance, in Taiwan, following the introduction of PCV13, a significant reduction in penicillin resistance was observed among IPD patients, from 82% in 2010 to 47% in 2020 (8). However, in New Zealand, reverting from PCV13 to PCV10 in 2017 led to an increase in serotype 19A resistance to 65% (6). High resistance rates are most frequently observed against penicillin and erythromycin (8, 11–14). Globally, the highest resistance rates to penicillin and erythromycin have been reported in serotypes 6B, 6A, 9V, 14, 15A, 19F, 19A, and 23F (13). The early and rapid administration of antibiotics is crucial to increase survival in IPD cases and the choice should be based on the local epidemiology of antibiotic susceptibility, among other factors (10). These trends underscore the importance of local epidemiology in guiding antibiotic therapy for IPD cases.

In Latvia, vaccination against pneumococcal disease was introduced into the NIP in 2010 with PCV7, later transitioning to PCV10 in 2012 and to PCV15 in 2024. The current immunization schedule includes 2 + 1 doses administered at 2, 4, and 12–15 months, and the vaccine is provided free of charge for all children. Recommendations for adult vaccination against pneumococcal infection only began in 2019, with vaccination being self-funded, even for those in high-risk groups.

The objective of our study is to determine the changes in *S. pneumoniae* antimicrobial resistance and its association with PCV10 serotypes in Latvia during the period from 2012 to 2022 based on surveillance data and to describe the changes in invasive *S. pneumoniae* isolates detected during the period from 2012 to 2022.

## Materials and methods

### Study population and design

We conducted a population-based cross-sectional study based on national IPD surveillance data over 11 years (2012–2022).

### Case definitions

IPD case in the study was defined according to the EU case definition, which was established in 2018 (15). All IPD diagnoses for surveillance data were coded according to ICD10 5-th version: B95.3, A40.3 for septicemia, G00.1, G00.2 for meningitis, and J14 for Invasive pneumococcal pneumonia.

### IPD surveillance in Latvia

All physicians should report IPD cases and all Laboratories should report all positive *S. pneumoniae* isolates from normally sterile sites to the Centre for Disease Prevention and Control (CDPC) of Latvia (4).

According to cabinet regulation No 7 “Procedures for Registration of Infectious Diseases,” laboratories, using the urgent reporting form provide information about *S. pneumoniae* antimicrobial susceptibility to the CDPC (16).

### Laboratory methods

#### Microbiological isolation and identification

The isolation of *S. pneumoniae* isolates was performed on onto tryptic soy agar plates supplemented with 5% sheep blood and incubated aerobically at 36 ± 1°C 24–48 h in 5% CO2-enriched air. Suspected *α*-haemolytic colonies were chosen for identification, colored by Gram, and tested for inhibition by optochin. Identification was performed in the VITEK-2 Compact analyzer.

#### Serotyping

Two methods were used to determine serotypes: latex agglutination and capsular sequence typing Latex agglutination was performed using Diagnostica ImmuLex™ Pneumotest Kit (Statens Serum Institut, Denmark). The mentioned Kit was intended for visual qualitative serogrouping and serotyping of *Streptococcus pneumoniae* by use of a rapid agglutination.

The rapid agglutination test is performed by mixing a drop of ImmuLex™ solution and a drop of pneumococcus culture solution on a reaction card. If the test is positive, agglutination will show within 10 s resulting in large visible aggregates. The aggregates consist of pneumococcal bacteria and latex particles from the ImmuLex™ solution. These aggregates are formed as a result of an antigen–antibody reaction between the pneumococcal capsule (antigen) and its homolog antibodies coated on the latex particles. No agglutination and no aggregation will show if the test is negative. The ImmuLex™ Pneumotest Kit identifies 92 pneumococcal serotypes using a Chessboard method.

Serotypes that were not possible to identify by latex agglutination were detected by the protocol for *Streptococcus pneumoniae* capsular sequence typing. The materials used for testing are DNA isolated from *S. pneumoniae* cultures or primary clinical material positive for *S. pneumoniae* DNA (e.g., cerebrospinal fluid). DNA isolation from cultures is performed using the boiling method or the commercial DNeasy Blood & Tissue Kit (QIAGEN). For DNA isolation from cerebrospinal fluid, we use the NUCLISENS EasyMAG system by bioMérieux (principle: DNA binding to magnetic particles coated with silica). The testing steps are: DNA isolation, amplification of the *wzh* Gene, PCR products are detected using electrophoresis in a 2% agarose gel with TAE buffer, the PCR products are purified using the ExoSap enzyme mixture, sequencing reaction [performed using the sanger method with two primers (forward and reverse) and BigDye v.3.1], purification of the sequencing reaction (carried out using an ethanol/sodium acetate mixture), capillary electrophoresis (the purified sequencing products are analyzed using the Applied Biosystems 3500 Series Genetic Analyzer), sequence Assembly (raw sequencing data are processed and assembled using SeqScape v.2.5 software), data analysis [the capsular type (and corresponding serotype) is determined using the *Streptococcus pneumoniae* CST Typing Tool, Version 0.0] (17).

#### Antimicrobial susceptibility testing

Agar disk diffusion method and E-test method were used for antibiotic susceptibility testing. According to the IPD surveillance framework and the European Centre for Disease Prevention and Control (ECDC) European Surveillance System (TESSy) reporting requirements, susceptibility testing results were available for the following antibiotics: penicillin, erythromycin, and cefotaxime/ceftriaxone. Antimicrobial susceptibility was determined based on the minimum inhibitory concentration (MIC) and results were interpreted according to the European Committee on Antimicrobial Susceptibility Testing (EUCAST) guidelines for *S. pneumoniae*.^1^

### Statistical analysis

We calculated age (grouped into four age groups: <1, 1–17, 18–64, ≥65) and sex specific IPD average incidence. Population estimation was provided by the Central Statistical Bureau of Latvia.

We calculated *S. pneumoniae* total resistance and separate resistance for penicillin, erythromycin, and cefotaxime/ceftriaxone. Resistance differences between penicillin, erythromycin, and cefotaxime/ceftriaxone were assessed by Cohran’s Q test.

For calculating *S. pneumoniae* resistance associations IPD clinical presentation, we divided cases into two groups: (1) meningitis – IPD cases clinically presented as septicemia with meningitis and meningitis itself, (2) septicemia – IPD cases clinically presented like pneumonia with septicemia and septicemia itself.

We conducted univariable analysis to identify risk factors associated with penicillin, erythromycin, and cefotaxime/ceftriaxone resistance in IPD cases using odds ratio (OR), 95% confidence interval (95%CI) and *p-value* for risk factors [*S. pneumoniae* serotype groups, age groups (0–17, 18–64, 65+), sex, clinical presentation (meningitis, septicemia)]. We looked also into individual serotype profiles and calculated OR, 95%CI and *p*-value for those with a 10% resistance proportion to penicillin, erythromycin and cefotaxime/ceftriaxone. We conducted logistic regression to develop multivariable model, using risk factors that had *p*-value <0.05 in univariable analysis, and the Akaike Information Criterion (AIC) to compare different models.

*Streptococcus pneumoniae* serotypes detected in IPD cases were grouped by vaccine constituent serotypes (PCV10, PCV13, PCV15, PCV20, and PPPV23) (Table 1) and analyzed only for serotyped cases (752/811).

**Table 1:** *Streptococcus pneumoniae* serotypes grouped by vaccine constituent serotypes.

*S. pneumoniae* serotype groups	*S. pneumoniae* serotypes within the groups
PCV10	1,4,5,6B, 7F, 9V, 14, 18C, 19F, 23F
PCV13nonPCV10	3, 19A, 6A
PCV15nonPCV13	22F, 33F
PCV20nonPCV15	8, 10A, 11A, 12F, 15B
PPPV23nonPCV20	20, 17F, 9N
NonVacc	Any other *S. pneumoniae* serotypes

Data analysis was performed using IBM SPSS Statistics (29.0.0.0) and Jamovi (2.3.28). A two-sided *p*-value of less than 0.05 was considered statistically significant result.

## Results

### Invasive pneumococcal disease cases, clinical presentation and *Streptococcus pneumoniae* serotypes

811 IPD cases were reported to CDPC during the study period (males, 60.2%). There were significant differences in IPD cases over the study period (chi2 = 56.6, *p* < 0.001).

**Figure 1:**
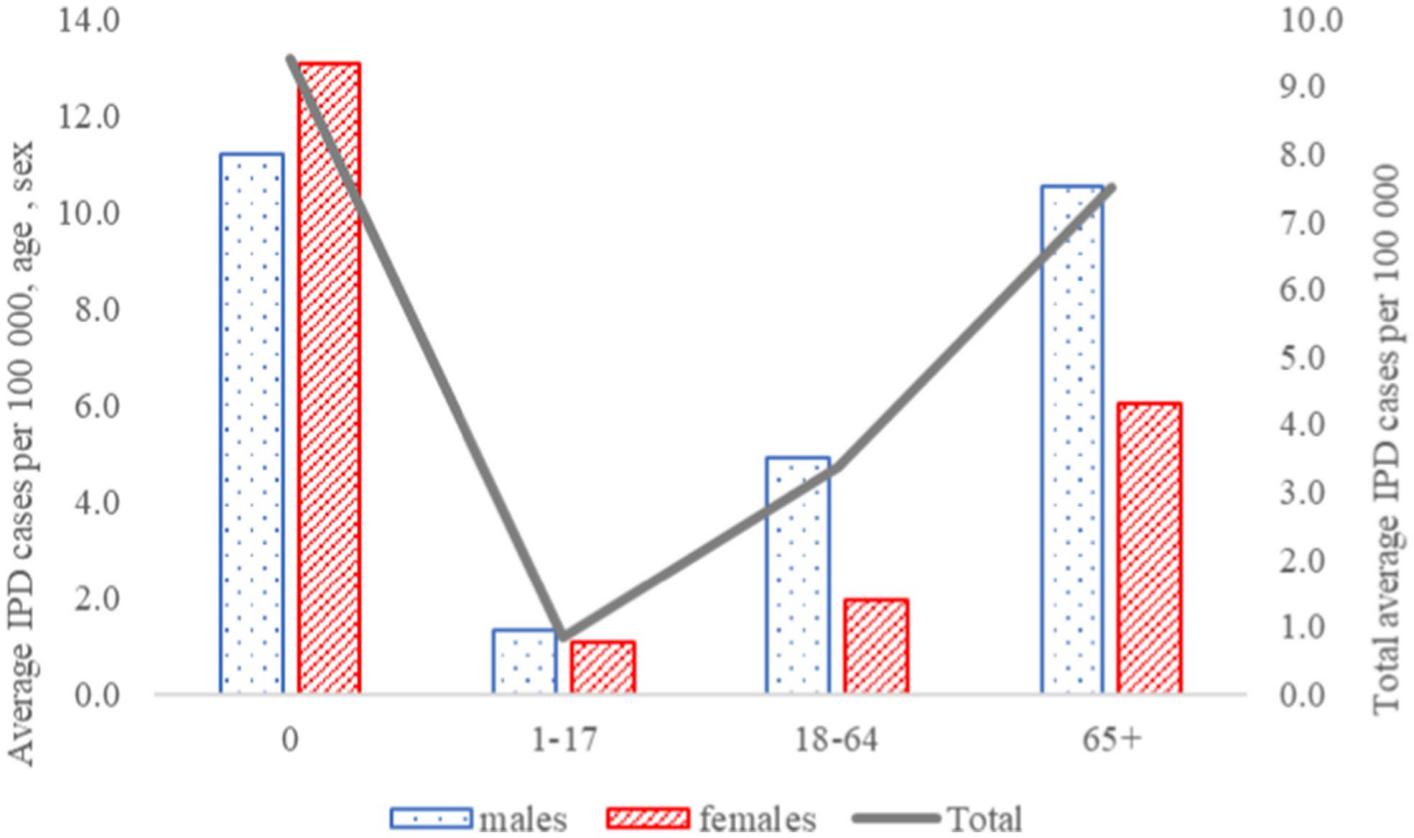
Average period incidence of Invasive pneumococcal disease by age and sex.

During the study period IPD incidence fluctuated from 2.7 cases per 100,000 inhabitants in 2012 to 6.7 cases per 100,000 inhabitants in 2022. Of the patients 1.6% (13/811) were infants (aged less than 1 year), 3.4% (28/811) were aged 1–17 years, 55.6% (451/811) were aged 18–64 years and 39.3% (319/811) were aged 65 or more. The highest mean annual incidence during the study period was reported in infants and those aged 65 or more (Figure 1).

Among all Invasive pneumococcal disease cases, clinical presentations with septicemia 84.4% (685/811) were the most common during the study period following those with meningitis 19% (158/811) and pneumonia 28.1% (228/811) (Table 2).

**Table 2:** Invasive pneumococcal disease clinical presentations in Latvia, 2012–2022.

	Septicemia with pneumonia		Meningitis		Meningitis with septicemia		Pneumonia		Septicemia		Total
	*n*	%	*n*	%	*n*	%	*n*	%	*n*	%	
2012	3	5.4	12	21.4	5	8.9	1	1.8	35	62.5	56
2013	28	50.0	10	17.9	2	3.6			16	28.6	56
2014	25	49.0	11	21.6	2	3.9			13	25.5	51
2015	16	18.4	15	17.2	3	3.4			53	60.9	87
2016	18	27.7	15	23.1	2	3.1	1	1.5	29	44.6	65
2017	16	21.3	15	20.0	7	9.3			37	49.3	75
2018	37	48.7	13	17.1	1	1.3			25	32.9	76
2019	42	50.6	11	13.3	3	3.6	1	1.2	26	31.3	83
2020	37	55.2	9	13.4		0.0			21	31.3	67
2021			4	5.7	3	4.3	2	2.9	61	87.1	70
2022			5	4.0	10	8.0	1	0.8	109	87.2	125
	222	27.4	120	14.8	38	4.7	6	0.7	425	52.4	811

92.7% (752/811) of isolates were serotyped. The most common serotypes were 3 and 19A, 17.4% (131/752) and 10.9% (82/752) respectively.

**Figure 2:**
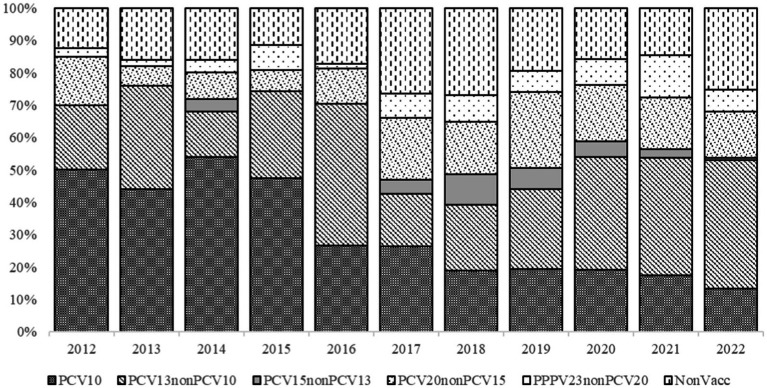
*Streptococcus pneumoniae* serotypes detected in IPD patients and grouped by vaccine constituent serotypes, 2012–2022.

PCV10 serotypes in IPD cases significantly decreased during the years, from 32% (18/56) in 2012 to 12% (16/125) in 2022 (*χ^2^* = 59.898, *p* < 0.001). However, PCV13 (*χ^2^* = 37.618, *p* < 0.001), PCV15 (*χ^2^* = 25.097, *p* = 0.005), and PPPV23 (*χ^2^* = 24.84, *p* = 0.006) serotypes were significantly increased over the study period (Figure 2).

### *Streptococcus pneumoniae* antimicrobial susceptibility in Invasive pneumococcal disease cases

Antimicrobial susceptibility testing for at least one of the three antibiotics (penicillin, erythromycin, or cefotaxime/ceftriaxone) was performed in 89.9% (729/811) of all reported IPD cases. Among these, susceptibility testing for erythromycin was conducted in 89.0% (722/811) of cases, for penicillin in 88.9% (721/811), and for cefotaxime/ceftriaxone in 82.6% (670/811). Overall, 7.5% (55/729) of tested IPD cases were classified as resistant to at least one of the three antibiotics. The specific proportions of non-susceptibility were: penicillin resistance was detected in 3.8% (28/721) of cases tested for this antibiotic, erythromycin resistance in 5.4% (39/722) of cases tested for erythromycin, and cefotaxime/ceftriaxone resistance in 1.2% (8/670) of cases tested for this antibiotic. Cefotaxime/Ceftriaxone demonstrates significantly lower resistance in comparison to penicillin (*p* = 0.001), and erythromycin (*p* < 0.001). There were no significant differences in penicillin and erythromycin resistance (*p* > 0.999).

There were no significant differences over the years in total resistance in IPD cases (*χ^2^* = 17.615, *p*-value = 0.062). However, erythromycin resistance demonstrated significant differences over the years (*χ^2^* = 21.786, *p* = 0.016), decreasing from 8.5% (4/47) in 2012 to 5.3% (6/114) in 2022. Penicillin resistance also decreased from 8.5% (4/47) in 2012 to 4.4% (5/114) but does not demonstrate any significant differences during the study period (*χ^2^* = 10.597, *p* = 0.390). Cefotaxime/Ceftriaxone resistance does not demonstrate any significant differences over the years (*χ^2^* = 7.237, *p* = 0.703). There were no cefotaxime/ceftriaxone resistant IPD cases in 2012, although in 2022 2.7% (3/114) IPD cases were resistant.

In univariable analysis, factors significantly associated with penicillin resistance were meningitis (OR = 2.94, *p* = 0.008, 95%CI 1.32–6.55), *S. pneumoniae* serotypes: 14 (OR = 16.91, *p* < 0.001, 95%CI 5.73–49.87), 23F (OR = 4.77, *p* = 0.018, 95%CI 1.31–17.35), 19F (OR = 3.95, *p* = 0.016, 95%CI 1.29–12.18) and PCV10 serotype group (OR = 3.91, *p* < 0.001, 95%CI 1.82–8.44). Factors significantly associated with erythromycin resistance in IPD cases were meningitis (OR = 2.11, *p* = 0.044, 95%CI 1.02–4.38), *S. pneumoniae* serotypes: 19A (OR = 4.61, *p* < 0.001, 95%CI 2.22–9.57), 14 (OR = 5.89, *p* = 0.003, 95%CI 1.83–18.99), 19F (OR = 4.59, *p* = 0.002, 95%CI 1.77–11.93), 15A (OR = 5.61, *p* = 0.011, 95%CI 1.48–21.27). Factors significantly associated with cefotaxime/ceftriaxone resistance were *S. pneumoniae* serotypes: 19A (OR = 5.32, *p* < 0.024, 95%CI 1.24–22.79), 19F (OR = 16.67, *p* < 0.001, 95%CI 3.75–74) (Table 3).

**Table 3:** Univariable factors associated with *Streptococcus pneumoniae* resistance in Invasive pneumococcal disease cases.

	Penicillin					Erythromycin					Cefotaxime/Ceftriaxone				
Factors	Resistance/cases	%	Resistance odds ratio (OR)	95%CI of RR	*p*-value of OR	Resistance/cases	%	Resistance odds ratio (OR)	95%CI of OR	*p*-value of OR	Resistance/cases	%	Resistance odds ratio (OR)	95%CI of OR	*p*-value of OR
Meningitis	10/158	6.3	**2.94**	**1.32–6.55**	**0.008**	11/158	7	**2.11**	**1.02–4.38**	**0.044**	2/158	1.3	1.84	0.37–9.3	0.455
Septicemia	18/647	2.8	0.36	**0.16–0.8**	0.013	28/647	4.3	0.5	0.24–1.04	0.065	6/647	0.9	0.57	0.11–2.9	0.506
Male sex	17/488	3.5	0.98	0.45–2.14	0.972	24/488	4.9	1.03	0.53–2.00	0.923	5/488	1	1.03	0.24–4.38	0.960
PCV10 serotypes	16/208	7.7	**3.91**	**1.817–8.11**	**<0.001**	14/208	6.7	1.58	0.8–3.1	0.178	3/208	1.4	2.67	0.43–16.59	0.292
PCV13nonPCV10 serotypes	6/219	2.7	0.68	0.24–1.70	0.41	13/219	5.9	1.31	0.66–2.61	0.436	3/219	1.4	1.47	0.35–6.19	0.603
PCV15nonPCV13 serotypes	0/23	0	NA			0/23	0	NA			1/23	4.3	5.42	0.63–46.53	0.123
PCV20nonPCV15 serotypes	1/107	0.9	0.21	0.029–1.59	0.133	0/107	0	NA			0/107	0	NA		
PPPV23nonPCV20 serotypes	0/14	0	NA			0/14	0	NA			0/14	0	NA		
NonVacc serotypes	4/181	2.2	0.56	0.19–1.64	0.289	9/181	4.9	1.03	0.48–2.22	0.939	1/181	0.5	0.49	0.06–4.06	0.514
Serotype 19A	5/82	6.1	1.96	0.72–5.33	0.185	12/82	14.6	**4.61**	**2.22–9.57**	**<0.001**	3/82	3.7	**5.32**	**1.24–22.79**	**0.024**
Serotype 14	6/18	33.3	**16.91**	**5.73–49.9**	**<0.001**	4/18	**22.2**	**5.89**	**1.83–18.99**	**0.003**	0/18	0	NA		
Serotype 23F	3/21	**14.3**	**4.77**	**1.31–17.35**	**0.018**	2/21	9.5	1.88	0.42–8.41	0.834	0/21	0	NA		
Serotype 19F	4/35	**11.4**	**3.95**	**1.29–12.18**	**0.016**	6/35	**17.1**	**4.59**	**1.77–11.93**	**0.002**	3/35	8.6	**16.67**	**3.75–74**	**<0.001**
Serotype 6B	2/20	10	3.25	0.71–14.9	0.128	1/20	5	1.17	0.15–9.1	0.879	0/20	0	NA		
Serotype 15A	1/14	7.1	2.1	0.26–16.76	0.483	3/14	21.4	**5.61**	**1.48–21.27**	**0.011**	1/14	7.1	8.45	0.96–74.67	0.055
Serotype 22F	0/23	0	NA			0/23	0	NA			1/23	4.3	5.42	0.63–46.53	0.123

Bold values are factors significantly associated with antimicrobial resistance.

Conducting multivariable analysis with logistic regression we found that *S. pneumoniae* serotypes 14 (adjusted(a)OR = 25.68, *p* < 0.001, 95%CI 8.05–84.96), 23F (aOR = 6.92, *p* = 0.005, 95%CI 1.78–27.01), 19F (aOR = 7.63, *p* < 0.001, 95%CI 2.29–25.35), and meningitis (aOR = 3.54, *p* = 0.005, 95%CI 1.48–8.49) were significantly associated with an increased likelihood of penicillin resistance. With an increased likelihood of erythromycin resistance were significantly associated with *S. pneumoniae* serotypes 19A (aOR = 9.63, *p* < 0.001, 95%CI 4.15–22.34), 14 (aOR = 13.26, *p* < 0.001, 95%CI 3.75–46.96), 19F (aOR = 11.47, *p* < 0.001, 95%CI 3.96–33.19), 15A (aOR = 10.03, *p* = 0.002, 95%CI 2.39–42.03), and meningitis (aOR = 2.91, *p* = 0.01, 95%CI 1.29–6.55). *S. pneumoniae* serotypes 19A (aOR = 12.81, *p* = 0.006, 95%CI 2.1–78.01) and 19F (aOR = 37.3, *p* < 0.001, 95%CI 5.94–234.21) were significantly associated with an increased likelihood of cefotaxime/ceftriaxone resistance.

## Discussion

The objective of the study was to determine the changes in *S. pneumoniae* antimicrobial resistance and its association with PCV10 serotypes in Latvia during the period from 2012 to 2022 using national surveillance data. Additionally, this study aims to describe the temporal trends in invasive *S. pneumoniae* isolates detected during the same period. Our findings demonstrate several key trends in antimicrobial resistance and provide important information about *S. pneumoniae* serotypes in Invasive pneumococcal disease cases.

Vaccination against pneumococcal infection was first introduced into the Latvian National Immunization Program in 2010 with the PCV7 vaccine. In 2012, PCV7 was replaced by PCV10, which remained in use until the end of 2023. Starting in early 2024, the vaccination program transitioned to the PCV15 vaccine. The introduction of PCV7, PCV10, and more recently PCV15 into the Latvian National Immunization Program has significantly influenced the *S. pneumoniae* serotype distribution in IPD cases. Our results indicate a decrease in the proportion of IPD cases caused by serotypes included in the PCV10 vaccine. However, our results show an increase in IPD cases caused by *S. pneumoniae* serotypes covered by PCV13, PCV15, and PPPV23 over the study period, suggesting possible serotype replacement. This is consistent with observations in other regions. In Canada where non-vaccine serotypes statistically significantly increased during the post-vaccination period (from 42% before vaccination to 73.3% during the PCV13 implementation period, *p* = 0.002) confirms that the introduction of PCV reduced the number of IPD cases caused by vaccine constituent *S. pneumoniae* serotypes. However, the increase in non-vaccine serotypes suggests serotype replacement (18). The same observations have been made in Spain after PCV13 introduction into the childhood immunization program. The number of IPD cases caused by PCV13 serotypes decreased by 88% (in children) and by 59% (in adults). However, the number of IPD cases caused by non-vaccine serotypes significantly increased (19). Austria also reports serotype 19A increasing from 3% to 6–7% in 2 years following PCV introduction (20) as well as Finland study indicates serotypes 19A (30%) and 3 (19%) as the most prevalent in IPD cases after PCV10 introduction (34). We observed in our study that introduction of PCV10 has reduced IPD cases caused by PCV10 vaccine serotypes, but PCV13nonPCV10 serotypes such as 3 and 19A become prevalent. This replacement has potential long-term implications such as severe IPD clinical presentation associated with 3 and 19A *S. pneumoniae* serotypes as well as serotype replacement possibly increasing the healthcare burden due to more severe clinical presentations and higher treatment costs for resistant infections. Latvia introduced PCV15 into the Latvian National Immunization Program which includes additional to PCV10 *S. pneumoniae* serotypes 3, 19A, 6A, 22F, 33F and could mitigate serotype replacement. To achieve this it is important to improve vaccine uptake rates in children and to prioritize for PCV vaccination high-risk groups, such as seniors and individuals with comorbidities.

*Streptococcus pneumoniae* serotype 3 was the most common serotype detected in IPD cases in Latvia during the study period. The same results were observed in the USA, where the most common *S. pneumoniae* serotypes identified in IPD patients were 3, 22F, 20, 35, 23A, 12.2%, 10.3%, 9.6, 9%, and 7.7%, respectively (21). Significant proportion of IPD cases (17.9%) were caused by serotypes not included in the vaccines. The emergence of non-vaccine serotypes (22F and 20) highlights the need for vaccines that cover a broader range of serotypes to reduce the burden of IPD (21, 33). As well Serbian study indicates serotype 3 (19.6%) as the most common in IPD cases (22).

The antibiotic consumption data in Latvia indicate one of the lowest reported in European Union (10.5 DDD per 1,000 inhabitants) (23) so our study results demonstrate relatively low antimicrobial resistance (7.5%) penicillin (3.8%), erythromycin (5.4%) and cefotaxime/ceftriaxone (1.2%) in IPD cases in comparison to other study results. Korean study results indicate that among IPD patients of all age groups, *S. pneumoniae* resistance to penicillin and ceftriaxone was 37.2% and 29.7%, respectively (24). Serbian study results shows high non-susceptibility percentage (40.4%) to erythromycin in IPD *S. pneumoniae* isolates (22). However World health organization reported penicillin resistance below 5% in 2020 in Austria, Czechia and the Netherlands while percentages equal to or above 25% were reported in Bosnia and Herzegovina, Cyprus, France, Iceland, Malta, Romania, Serbia and Turkey (25). In other countries, the rate of *S. pneumoniae* resistance to ceftriaxone among IPD patients was low: Japan (8.4%), South Africa (8.0%), and the USA (8.7%) (26, 27).

Our analysis shows that while penicillin and cefotaxime/ceftriaxone resistance trends did not demonstrate significant differences, there were significant differences in erythromycin resistance in IPD cases.

Multivariable logistic regression analysis revealed that *S. pneumoniae* PCV10 vaccine serotypes (particularly 14, 23F, 19F) detected in IPD patients and meningitis were strongly associated with an increased likelihood of penicillin resistance. Our findings indicate that patients with meningitis are over three times more likely to have penicillin resistant *S. pneumoniae* compared to other IPD cases. Other study results demonstrate that penicillin resistance in meningitis-causing *S. pneumoniae* strains has been noted as a critical concern due to the limited ability to achieve sufficient drug concentrations in the cerebrospinal fluid (28). Penicillin-resistant pneumococcal meningitis presents a significant clinical challenge. The study findings conducted in Spain demonstrate pneumococcal meningitis caused by penicillin-resistant strains significant association with mortality. Effective treatment is complicated by the blood–brain barrier, which limits antibiotic penetration into the cerebrospinal fluid. This is particularly problematic for resistant strains, requiring higher doses or combination therapies to achieve therapeutic levels. However, implementing high-dose cefotaxime requires careful monitoring for adverse effects, particularly in patients with comorbidities or in seniors (29). It is important to monitor not only meningitis caused by penicillin-resistant *S. pneumoniae* serotypes, but all reported IPD cases monitor for antimicrobial susceptibility and broader spectrum of antibiotics for susceptibility testing by enhancing IPD surveillance system. This surveillance helps in identifying emerging resistance trends, enabling timely updates to treatment guidelines. Meningitis and *S. pneumoniae* serotypes 19A (PCV13nonPCV10 vaccine constituent serotype), 14, 19F, and 15A (NonVacc serotype) also demonstrated a significant association with erythromycin resistance.

Further studies will cover comorbidities and other clinical factors for more comprehensive antimicrobial resistance analysis.

This indicates that vaccine serotype-specific resistance should be closely monitored to inform treatment guidelines and vaccine policy adjustments. Worldwide, the highest resistance rates to penicillin and erythromycin were found in serotypes 6B, 6A, 9V, 14, 15A, 19F, 19A, and 23F (12, 30), where 6B, 9V, 14, 19F, 23F are PCV10 vaccine serotypes.

Underreporting in a surveillance system and possible under diagnosis are likely to be study limitations. There are no clear guidelines for hospital laboratories regarding sample logistics. Some hospitals identify *S. pneumoniae* and test for antimicrobial susceptibility in their labs before sending the isolates to the National Reference Laboratory for further examination. There is a lack of information about the validation of identification and antimicrobial susceptibility tests in regional hospital laboratories. Moreover, PCR is not performed in these regional labs. Some hospitals directly send blood, CSF, or other sterile site samples to the NRL for *S. pneumoniae* identification, serotyping, and antimicrobial susceptibility detection, which prefers *S. pneumoniae* isolation over PCR. This approach may lead to an underestimation of cases.

Our study findings may have influenced by underreporting and under diagnosis described. This limitation possibly introduce bias, particularly in assessing the true burden of antimicrobial resistance. The selection of antibiotics for susceptibility testing reflects the limitations of the Latvian IPD surveillance system. Antimicrobial susceptibility data from Invasive pneumococcal disease patients reported to the surveillance system are collected in accordance with ECDC TESSy reporting requirements and submitted to the Centre for Disease Prevention and Control of Latvia (CDPC), which further disseminate surveillance data. TESSy mandates that countries report susceptibility to three antibiotics: penicillin, erythromycin, and cefotaxime/ceftriaxone. To mitigate these limitations in future studies we suggest following potential improvements to the IPD surveillance system: to incorporate routine PCR testing, standardize sample logistics, include all tested antimicrobial susceptibility notification in the case based surveillance and establish a centralized reporting platform to enhance case capture.

We suggest to conduct IPD surveillance system sensitivity analysis to detect possible underreporting rates and evaluate their potential effect on the IPD incidence and antimicrobial resistance findings. However our study reports an increase in IPD incidence in 2022. Similar increase in IPD incidence were observed in other countries following the easing of COVID-19 pandemic restrictions (31). It is important to continue monitor IPD incidence trends and describe potential factors contributing to these fluctuations.

Our findings are important for public health strategies, underlying the dynamic IPD and *S. pneumoniae* epidemiology. Surveillance of *S. pneumoniae* serotypes distribution and antimicrobial resistance patterns is important to vaccination programs. The introduction of the PCV15 into NIP in 2024 is an important step in the prevention of Invasive pneumococcal disease. PCV15 covers additional serotypes, including 22F and 33F, which are increasingly implicated in non-vaccine serotype replacements observed globally. A dynamic transmission model specific to Germany predicted that the introduction of PCV15 could lead to a reduction in IPD incidence by approximately 6% over 10 years compared to continued use of PCV13 (32). PCV15 introduction into NIP potentially will provide coverage against additional serotypes covered by this vaccine. Future studies should investigate the long-term PCV15 impact on *S. pneumoniae* serotype replacement dynamics and antimicrobial resistance. Additionally, compensation for vaccination against pneumococcal disease for high-risk group adults could further reduce the IPD burden and mitigate the spread of resistant *S. pneumoniae.*

This study provide national overview of *S. pneumoniae* serotypes identified in patients with IPD from 2012 to 2022, analyzing serotype replacement in relation to the pneumococcal vaccines available in the country, as well as antimicrobial resistance trends. Future studies will focus on analyzing changes in IPD incidence and regional stratification of IPD cases.

## Conclusion

Overall, our study provides a comprehensive insight into the epidemiological diversity of *S. pneumoniae* isolates causing Invasive pneumococcal disease in Latvia in 11 year period. The most common serotypes were 3 and 19A. Our data demonstrate low *S. pneumoniae* antimicrobial resistance and resistance decreasing to erythromycin and penicillin in IPD cases over the years. Moreover, meningitis as IPD clinical presentation was significantly associated with penicillin and erythromycin resistance.

Further studies to monitor PCV15 impact on *S. pneumoniae* serotypes circulation are useful, as well as continuous *S. pneumoniae* serotypes antimicrobial resistance surveillance is needed to observe further trends in serotypes replacement.

## Data Availability

The original contributions presented in the study are included in the article/supplementary material, further inquiries can be directed to the corresponding author.

## Footnotes

^1^ https://mic.eucast.org/search/?search%5Bmethod%5D=mic&search%5Bantibiotic%5D=-1&search%5Bspecies%5D=501&search%5Bdisk_content%5D=-1&search%5Blimit%5D=50

## References

[1] Houseman C Chapman KE Manley P Gorton R Wilson D Hughes GJ. Decreasing case fatality rate following invasive pneumococcal disease, north East England, 2006-2016. Epidemiol Infect. (2019) 147:e175. doi: 10.1017/S0950268819000657, PMID: 31063115 PMC6518772

[2] Scelfo C Menzella F Fontana M Ghidoni G Galeone C Facciolongo NC. Pneumonia and invasive pneumococcal diseases: the role of pneumococcal conjugate vaccine in the era of multi-drug resistance. Vaccines. (2021) 9:420. doi: 10.3390/vaccines9050420, PMID: 33922273 PMC8145843

[3] World Health Organization. WHO position paper on pneumococcal conjugate vaccines in infants and children under 5 years of age. (2019). Available online at: https://apps.who.int/iris/bitstream/handle/10665/310968/WER9408.pdf?ua=1.

[4] Savrasova L Krumina A Cupeca H Zeltina I Villerusha A Grope I. Invasive Pneumococcal Disease in Latvia in PCV10 Vaccination Era, 2012-2018. Front Pediatr. (2021) 9:532489. doi: 10.3389/fped.2021.532489, PMID: 34692599 PMC8529945

[5] Drijkoningen JJ Rohde GG. Pneumococcal infection in adults: burden of disease. Clin Microbiol Infect. (2014) 20:45–51. doi: 10.1111/1469-0691.12461, PMID: 24313448

[6] Anglemyer A McNeill A DuBray K Sonder GJB Walls T. Invasive pneumococcal disease: concerning trends in serotype 19A notifications in New Zealand. Clin Infect Dis. (2022) 74:1859–61. doi: 10.1093/cid/ciab766, PMID: 34480534

[7] Dai G Wang T He Y Jiang W Sun H Chen Z. Antimicrobial susceptibility and serotype distribution of *Streptococcus pneumoniae* isolates among children in Suzhou, China. Transl Pediatr. (2023) 12:2203–12. doi: 10.21037/tp-23-547, PMID: 38197098 PMC10772826

[8] Huang H Lin C-Y Chiu N-C Huang DT-N Huang C-Y Chi H. Antimicrobial susceptibility and serotype replacement of *Streptococcus pneumoniae* in children before and after PCV13 introduction in Taiwan. J Microbiol Immunol Infect. (2023) 56:299–310. doi: 10.1016/j.jmii.2022.08.018, PMID: 36127232

[9] Reis JN Azevedo J de Oliveira AML Menezes APDO Pedrosa M dos Santos MS. Long-term surveillance of invasive pneumococcal disease: the impact of 10-valent pneumococcal conjugate vaccine in the metropolitan region of Salvador, Brazil. Vaccine. (2024) 42:591–7. doi: 10.1016/j.vaccine.2023.12.055, PMID: 38184393 PMC10872423

[10] Ben-Shimol S Givon-Lavi N Greenberg D Stein M Megged O Bar-Yochai A. Impact of pneumococcal conjugate vaccines introduction on antibiotic resistance of *Streptococcus pneumoniae* meningitis in children aged 5 years or younger, Israel, 2004 to 2016. Euro Surveill. (2018) 23:1800081. doi: 10.2807/1560-7917.ES.2018.23.47.1800081, PMID: 30482264 PMC6341944

[11] Fu J Yi R Jiang Y Xu S Qin P Liang Z. Serotype distribution and antimicrobial resistance of *Streptococcus pneumoniae* causing invasive diseases in China: a meta-analysis. BMC Pediatr. (2019) 19:424. doi: 10.1186/s12887-019-1722-1, PMID: 31711442 PMC6844036

[12] Hascelik G Soyletir G Gulay Z Sancak B Yaman A Gurler N. Serotype distribution of *Streptococcus pneumoniae* and pneumococcal vaccine coverage in adults in Turkey between 2015 and 2018. Ann Med. (2023) 55:266–75. doi: 10.1080/07853890.2022.2160877, PMID: 36579976 PMC9809394

[13] Sandoval MM Ruvinsky S Palermo MC Alconada T Brizuela ME Wierzbicki ER. Antimicrobial resistance of *Streptococcus pneumoniae* from invasive pneumococcal diseases in Latin American countries: a systematic review and meta-analysis. Front Public Health. (2024) 12:1337276. doi: 10.3389/fpubh.2024.1337276, PMID: 38317800 PMC10839967

[14] Valenciano SJ Moiane B Lessa FC Chauque A Massora S Pimenta FC. Effect of 10-Valent pneumococcal conjugate vaccine on *Streptococcus pneumoniae* nasopharyngeal carriage among children less than 5 years old: 3 years Post-10-Valent pneumococcal conjugate vaccine introduction in Mozambique. J Pediatric Infect Dis Soc. (2021) 10:448–56. doi: 10.1093/jpids/piaa132, PMID: 33245124

[15] Commission Implementing Decision (EU) 2018/945 on the communicable diseases and related special health issues to be covered by epidemiological surveillance as well as relevant case definitions. (2018). Available online at: https://eur-lex.europa.eu/eli/dec_impl/2018/945/oj

[16] Republic of Latvia Cabinet Regulation No, 7 Adopted 5 January 1999 procedures for registration of infectious diseases. Riga: Latvijas Vēstnesis. 5/6 (1465/1466), 08,01,1999 (1999) legislation act, Cabinet regulation.

[17] Elberse KE van de Pol I Witteveen S van der Heide HG Schot CS van Dijk A. Population structure of invasive *Streptococcus pneumoniae* in the Netherlands in the pre-vaccination era assessed by MLVA and capsular sequence typing. PLoS One. (2011) 6:e20390. doi: 10.1371/journal.pone.0020390, PMID: 21637810 PMC3102707

[18] Murad Y Hung T-Y Sadarangani M Morris SK Le Saux N Vanderkooi OG. Clinical presentations and outcomes of children in Canada with recurrent invasive pneumococcal disease from the IMPACT Surveillance network. Pediatr Infect Dis J. (2022) 41:E166–71. doi: 10.1097/INF.0000000000003454, PMID: 35093996 PMC8920017

[19] de Miguel S Domenech M Gonzalez-Camacho F Sempere J Vicioso D Sanz JC. Nationwide trends of invasive pneumococcal disease in Spain from 2009 through 2019 in children and adults during the pneumococcal conjugate vaccine era. Clin Infect Dis. (2021) 73:e3778–87. doi: 10.1093/cid/ciaa1483, PMID: 32990303

[20] Paulke-Korinek M Kollaritsch H Kundi M Schmidle-Loss B Zwazl I Laaber B. Characteristics of invasive pneumococcal disease in hospitalized children in Austria. Eur J Pediatr. (2014) 173:469–76. doi: 10.1007/s00431-013-2193-2, PMID: 24221605

[21] Schulz PS Moore SE Smith D Javed J Wilde AM. Missed pneumococcal vaccination opportunities in adults with invasive pneumococcal disease in a community health system. Open Forum Infect Dis. (2022) 9:ofac075. doi: 10.1093/ofid/ofac075, PMID: 35308484 PMC8926003

[22] Opavski N Jovicevic M Kabic J Kekic D Vasiljevic Z Tosic T. Serotype distribution, antimicrobial susceptibility and molecular epidemiology of invasive *Streptococcus pneumoniae* in the nine-year period in Serbia. Front Microbiol. (2023) 14:1244366. doi: 10.3389/fmicb.2023.1244366, PMID: 37670985 PMC10475725

[23] European Centre for Disease Prevention and Control. Country mission Latvia: antimicrobial resistance. Stockholm: ECDC (2013).

[24] Kim GR Kim EY Kim SH Lee HK Lee J Shin JH. Serotype distribution and antimicrobial resistance of *Streptococcus pneumoniae* causing invasive pneumococcal disease in Korea between 2017 and 2019 after introduction of the 13-Valent pneumococcal conjugate vaccine. Ann Lab Med. (2023) 43:45–54. doi: 10.3343/alm.2023.43.1.45, PMID: 36045056 PMC9467834

[25] WHO Regional Office for Europe/European Centre for Disease Prevention and Control. Antimicrobial resistance surveillance in Europe 2022–2020 data. Copenhagen: WHO Regional Office for Europe (2022).

[26] Kawaguchiya M Urushibara N Aung MS Kudo K Ito M Sumi A. Clonal lineages and antimicrobial resistance of nonencapsulated *Streptococcus pneumoniae* in the post-pneumococcal conjugate vaccine era in Japan. Int J Infect Dis. (2021) 105:695–701. doi: 10.1016/j.ijid.2021.02.109, PMID: 33676003

[27] Lee YJ Huang Y-T Kim SJ Kerpelev M Gonzalez V Kaltsas A. Trends in invasive pneumococcal disease in cancer patients after the introduction of 7-valent pneumococcal conjugate vaccine: a 20-year longitudinal study at a major urban Cancer center. Clin Infect Dis. (2018) 66:244–53. doi: 10.1093/cid/cix739, PMID: 29020313 PMC5850633

[28] Koelman DLH Brouwer MC Ter Horst L Bijlsma MW van der Ende A van de Beek D. Pneumococcal meningitis in adults: a prospective nationwide cohort study over a 20-year period. Clin Infect Dis. (2022) 74:657–67. doi: 10.1093/cid/ciab477, PMID: 34036322 PMC8886910

[29] Cabellos C Guillem L Pelegrin I Tubau F Ardanuy C Gudiol F. Penicillin- and cephalosporin-resistant pneumococcal Meningitis: treatment in the real world and in guidelines. Antimicrob Agents Chemother. (2022) 66:e0082022. doi: 10.1128/aac.00820-22, PMID: 36326246 PMC9764967

[30] Desmet S Theeten H Laenen L Cuypers L Maes P Bossuyt W. Characterization of emerging serotype 19A pneumococcal strains in invasive disease and carriage, Belgium. Emerg Infect Dis. (2022) 28:1606–14. doi: 10.3201/eid2808.212440, PMID: 35876488 PMC9328928

[31] Covadonga P-G Sempere J Miguel S Hita S Úbeda A Vidal EJ. Surveillance of invasive pneumococcal disease in Spain exploring the impact of the COVID-19 pandemic (2019-2023). J Infect. (2024) 89:106204. doi: 10.1016/j.jinf.2024.106204 38906265

[32] Horn M Theilacker C Sprenger R Mahar E Schiffner-Rohe J Pletz MW. Mathematical modeling of pneumococcal transmission dynamics in response to PCV13 infant vaccination in Germany predicts increasing IPD burden due to serotypes included in next-generation PCVs. PLoS One. (2023) 18:e0281261. doi: 10.1371/journal.pone.0281261 36791091 PMC9931105

[33] Grant LR Slack MPE Theilacker C Vojicic J Dion S Reinert R-R. Distribution of serotypes causing invasive pneumococcal disease in children from high-income countries and the impact of pediatric pneumococcal vaccination. Clin Infect Dis. (2023) 76:e1062–70. doi: 10.1093/cid/ciac475, PMID: 35789262 PMC9907512

[34] Jokinen J Rinta-Kokko H Siira L Palmu AA Virtanen MJ Nohynek H. Impact of ten-valent pneumococcal conjugate vaccination on invasive pneumococcal disease in Finnish children – a population-based study. PLoS One. (2015) 10:e0120290. doi: 10.1371/journal.pone.0120290, PMID: 25781031 PMC4364013

